# Placental damage in pregnancies with systemic lupus erythematosus: A narrative review

**DOI:** 10.3389/fimmu.2022.941586

**Published:** 2022-08-17

**Authors:** Aleida Susana Castellanos Gutierrez, Francesc Figueras, Diana M. Morales-Prieto, Ekkehard Schleußner, Gerard Espinosa, Núria Baños

**Affiliations:** ^1^ BCNatal, Barcelona Center for Maternal-Fetal and Neonatal Medicine (Hospital Clínic and Hospital Sant Joan de Déu), Institut Clínic de Ginecologia, Obstetrícia i Neonatologia Fetal i+D Fetal Medicine Research Center, Barcelona, Spain; ^2^ Placenta Lab, Department of Obstetrics, Jena University Hospital, Jena, Germany; ^3^ Institut d’Investigacions Biomèdiques Pi i Sunyer (IDIBAPS), Barcelona, Spain; ^4^ Department of Autoimmune Diseases, Hospital Clinic, Barcelona, Spain

**Keywords:** systemic lupus erythematosus, placenta, histopathology, antiphospholipid antibodies, complement system, neutrophil extracellular traps, cytokines

## Abstract

Systemic lupus erythematosus (SLE) is a chronic inflammatory autoimmune disease of unknown cause, which mainly affects women of childbearing age, especially between 15 and 55 years of age. During pregnancy, SLE is associated with a high risk of perinatal morbidity and mortality. Among the most frequent complications are spontaneous abortion, fetal death, prematurity, intrauterine Fetal growth restriction (FGR), and preeclampsia (PE). The pathophysiology underlying obstetric mortality and morbidity in SLE is still under investigation, but several studies in recent years have suggested that placental dysfunction may play a crucial role. Understanding this association will contribute to developing therapeutic options and improving patient management thus reducing the occurrence of adverse pregnancy outcomes in this group of women. In this review, we will focus on the relationship between SLE and placental insufficiency leading to adverse pregnancy outcomes.

## 1 SLE generalities

### 1.1 Epidemiology

The reported female-to-male ratio of SLE prevalence is 9:1. SLE is manifesting between the second and fourth decade of life, affecting women of childbearing age. In the general female population, SLE has a prevalence of approximately 1:1000. However, some differences between races have been reported, e.g., SLE is less frequent and less severe in white than black or Asian women (1).

### 1.2 Etiology

SLE is an autoimmune disease in which organs, tissues, and cells are damaged by the adhesion of various autoantibodies and immune complexes. The etiology of SLE is largely unknown, but some factors are associated with the presence of the disease, such as dysregulation of the immune system, genetic, hormonal, and environmental factors.

#### 1.2.1 Hormonal dysfunction

The hormonal role is deduced because up to 90% of the cases correspond to reproductive age women. Furthermore, the risk of the disease is increased in women receiving hormone replacement therapy with conjugated estrogens and progesterone. Still, it is not possible to date establish the role of hormones in the onset of SLE (1–4).

#### 1.2.2 Genetic factors

In SLE, genetic factors play a role in the susceptibility to develop it but are insufficient to cause it. The coincidence rate in monozygotic twins is approximately 25% and 2% in dizygotic twins. Several genotypes have been identified in families with multiple members with SLE (5). Different studies observed the association of SLE with Human Leukocyte Antigen (HLA) class 2 antigens (HLA-DR2 and DR3) in both white and black ethnicities; as well as with hereditary diseases due to complement deficiency: C1r, C1s, C1, INH, C4, C2, C5, and C8, mainly with C2 lack. Partial C2 deficiency in heterozygotes is also more frequent, 6% in SLE vs. 1% on average (6).

##### 1.2.2.1 HLA genes in SLE

The first genetic association described for SLE was with the HLA region on chromosome 6p21.3 (7), which encodes more than 200 genes, many of them with known immunologic functions. The HLA region is subdivided into the class I and class II regions, which contain genes encoding glycoproteins that process and present peptides for recognition by T cells, and the class III region, which includes other important immune genes (such as TNF, C2, C4A, C4B, and CFB). However, the available evidence suggests that genetic variants of HLA class II genes (such as HLA-DR2 and HLA-DR3) and class III genes (such as MSH5 and SKIV2L), in particular, predispose an individual to SLE (8).

##### 1.2.2.2 Non-HLA genes in SLE

During the last few years, thanks to advances in genetics, different genes associated with SLE development have been studied. Genome wide association studies have identified genetic and transcriptional abnormalities in key molecules directly involved in the type I Interferon (IFN) signaling pathway, namely TYK2, STAT1 and STAT4 and IRF5. Also, common polymorphisms in or near TLR7, which are related to the synthesis of interferons, seems to be involved with the development of the disease. In fact, recent evidence confirmed human SLE caused by a TLR7 gain-of-function variant (9–11).

The transcription factor IRF5 has been genetically linked to SLE and many other autoimmune diseases, suggesting a central role of this protein in regulating the immune response; on the other hand, it holds the expression of IFN-dependent genes, inflammatory cytokines, and genes involved in apoptosis (8).

#### 1.2.3 Environmental factors

Several drugs may induce a variant of SLE, mainly quinidine, procainamide, and hydralazine (12). In this form of SLE, dermatological and articular manifestations are frequent, unlike renal and neurological manifestations. Other potential factors are ultraviolet radiation as a trigger for specific skin lesions and diets rich in fats, which can modify the immune response (12).

### 1.3 SLE and reproduction

#### 1.3.1 Fertility

Fertility is not affected by SLE in the absence of lupus activity (13). However, SLE is associated with the age at which women decide or can become mothers. In general, SLE patients tend to be older when it comes to becoming pregnant, as they are advised to plan conception only after six months without flares (14–16). The effect of aging on female fertility is detrimental to successful conception. In addition, organ involvement and the treatment used to treat SLE activity can lead to abnormal ovarian function or ovarian failure, making it difficult for SLE patients to become pregnant. For example, alkylating agents such as cyclophosphamide can develop menstrual irregularities and premature ovarian failure; these side effects are also related to the dose administered and the age of the patients (16, 17). Therefore, patients should be adequately counseled regarding the age of seeking pregnancy (tendency to postpone childbearing) and healthy lifestyles (exercise, tobacco use, alcohol consumption, avoiding overweight) (17).

#### 1.3.2 Adverse pregnancy outcomes

Despite improved treatment, pregnancies of women with SLE continue to have higher perinatal morbidity compared to the general population (1, 3). According to the literature, one the most frequent fetal complications are spontaneous abortion (16.0%), one-third of pregnancies in patients with SLE will end by cesarean section, 39.4% will have a preterm delivery, 2-8% will have PE and 12% will have FGR (18, 19). On average, the elevated risk of prematurity in patients with SLE is related to premature rupture of membranes (PROM). A part of preterm deliveries are caused by iatrogenesis due to maternal or fetal condition deterioration (20). Further, the probability of preterm delivery increases by up to two-thirds in women with moderate to severe SLE activity during this period (21).

PE is more frequent in SLE women who have suffered from lupus nephritis and renal insufficiency at the time of conception. Other risk factors associated with the occurrence of PE in SLE patients are a previous history of PE, first gestation, maternal age ≥ 40 years, active lupus at the time of conception, high levels of anti-dsDNA antibodies, anti-ribonucleoprotein antibodies positive, low levels of serum complement, obesity (BMI ≥ 35 kg/m2), pre-existing hypertension, and diabetes (22). In addition, is relevant to mention that pregnant patients with SLE who developed PE, showed raised levels of IFN-α activity before the onset of clinical symptoms, which could not be explained by higher levels of disease activity or autoantibodies. It has also been shown that type I interferons suppress the production of angiogenic factors (particularly fibroblast growth factor, interleukin-8, and VEGF) and trigger anti-angiogenic gene expression, lessening endothelial cell migration and suppressing endothelial progenitor cell proliferation (23).

Pregnancy loss is a relatively frequent event observed in 16% of pregnancies in healthy women. However, in those with SLE, the rate of miscarriage increases to 21.7%, reaching as much as 50% in women with lupus nephritis at the expense of fetal death during the second trimester (5). The incidence of fetal loss in SLE will rise by the existence of renal or neurological involvement, and the presence of certain antibodies such as anti-Ro/SSA anti-La/SSB, anti-thyroglobulin and especially antiphospholipid antibodies (aPLs) (24). Anti-Ro/SSA anti-La/SSB have been found in 10% of SLE patients. They are strongly associated with congenital heart block as they pass the placental barrier after 16 weeks, regardless of maternal disease activity or classification. The risk of developing congenital heart block is 1.5-2%, in case of a history of non-cardiac neonatal lupus, the risk is up to 15%, and 20% in the case of a previous child with congenital heart block (risk rising to 40-45% after two last affected children). In addition, it can condition the appearance of hydrops in 40-60% of cases, with a risk of perinatal mortality estimated at 45-50% and the need for a postnatal pacemaker of > 80% (19, 25).

Other complications described during pregnancy such as stroke, thrombotic disease (deep vein thrombosis, pulmonary embolism), infections, bleeding, thrombocytopenia, need for transfusion, and even maternal death are elevated in patients with SLE (21, 22).

## 2 Placental insufficiency and SLE

The formation of the placenta begins as soon as 5-6 days after fertilization when the trophoblast cells meet the endometrium. Subsequently, trophoblast cells invade and proliferate within maternal tissues, accessing the uterine vessels and causing their remodelation to provide sufficient oxygen and nutrients for the developing fetus (19). Placental failure is related to an inadequate invasion of the uterine arteries by the trophoblast cells, resulting in the maintenance of high vascular resistances with the consequent production of defective, turbulent, and high-velocity flow within the intervillous space. This puts pressure on the chorionic villi and impairs nutrient absorption as well as oxygen exchange causing respectively fetal malnutrition and chronic fetal hypoxia (26, 27). Chronic hypoxia leads to inadequate formation of chorionic villi in the early stages of placental development, which, in turn, worsens oxygen hypoperfusion, setting in motion an aggravating cycle throughout gestation (19, 28).

Patients with SLE have a higher risk of developing adverse pregnancy outcomes mainly related to an impaired placentation. Therefore, understanding how SLE affects the placenta is fundamental to improve patient follow-up and treatments, thus reducing pregnancy complications. Below we summarize the histopathological findings and some of the proposed mechanisms that may explain the association between SLE and the development of pregnancy complications.

### 2.1 Placental histopathological findings

Only a handful of studies have attempted to identify the placental lesions in patients with SLE. These changes include mostly abnormalities of placental vascularity and coagulation, e.g., extensive infarction that has been related to preterm delivery and fetal death. Decidual vasculopathy is observed in up to 17% of previously studied placentas and is related to preterm delivery (29–31). Placental abruption and decidual thrombi have been associated with fetal death (32, 33). Less frequently, inflammatory lesions, chronic inflammation, decidual vasculitis, and chronic villitis have been described in 28% of placentas, related to low placental weight (30, 31, 34). In addition, decreased placental weight is a common feature of SLE (35).

In another study of placentas three histologic patterns were reported most frequently: coagulation-related lesions, placental villous and vascular damage, and chronic inflammation in women with autoantibodies and preterm delivery (36). Because of heterogeneous patterns observation, the study conclude that placental damage seems to be mediated by a pathophysiological processes. Uteroplacental vascular pathology and villous lesions are thought to be secondary to physiological changes in the myometrial portion of the spiral arteries, which may reflect abnormal immunological recognition of trophoblastic antigens (36). Coagulation-related lesions could be cause as a secondary to a thrombotic effect of aPLs on placental vessels. Indeed, the presence of aPLs in a population of women with SLE, was associated with an increased rate of placental infarcts and ischemic villous lesions, but not decidual vasculopathy decidual or fetal thrombi, chronic villitis, or perivascular fibrin deposition. Chronic inflammatory lesions may also reflect vasculitis, a common SLE feature (36).

**Figure 1:**
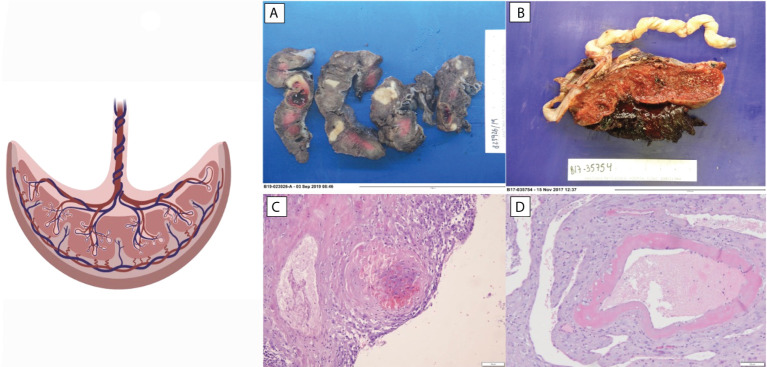
Histopathologic changes observed in placentas of patients with SLE. **(A)** Extensive infarction. **(B)** Abruptio placenta. **(C)** Decidual vasculopathy. **(D)** Decidua thrombi.

Reduced placenta weight is a surrogate marker of maternal malperfusion and a predictor for stillbirth (37). The increased risk of stillbirth related to low placental weight suggests that a decreased placental surface area for gas and nutrient exchange may lead to fetal compromise (38, 39). In a prospective study, SLE placentas were found smaller and having lower weight than healthy pregnant women placentas, and these findings correlated with SLE activity during pregnancy, thrombocytopenia, and hypocomplementemia (40, 41). Chronic villitis of unknown implied etiology (VUE) was also associated with decreased placental weight in placentas from SLE patients. The study observed that the percentage of placentas presenting this histological finding was more than three times higher than control population group (**Figure 1**). The appearance of VUE seems to be related to SLE. Other studies have observed that VUE manifests late in pregnancy (third trimester) and suggests that it is an immunologic lesion mediated by maternal inflammatory cells (31, 42).

## 3 Molecular mechanisms in SLE

Several mechanisms have been proposed to explain and understand the histopathological changes in SLE placentas. These include the deposition of complement and immunoglobulins in the walls of the decidual blood vessels (30), decidual thrombosis due to aPLs (1, 35), and alterations of the immune system including T-lymphocyte–mediated inflammatory reaction (33). In the following sections, these mechanisms will be addressed in detail.

### 3.1 Complement system

The complement system is composed by more than 30 proteins present in the blood, tissues and other proteins anchored to the surface of cells. The main functions of the complement system are protection against infections, removal of certain substances (such as damaged cells, microbes, or immune complexes), and assisting in the modulation of adaptive immune responses. Bacterial infections and autoimmune diseases are the most frequent clinical conditions associated with complement deficiencies. The most crucial complement elements involved in SLE development are C1, C3, and C4 (42, 43).

During embryo implantation complement activity is elevated, which is essential to promote trophoblast invasion of the decidua. After implantation, complement levels physiologically decrease but they remain elevated in miscarriage cases (44). Adequate complement system activation is critical to maintaining a normal pregnancy; unregulated complement activation represents a risk for damage to the integrity of the placental barrier and can have devastating effects on both fetal and maternal well-being. In fact, hypocomplementemia during gestation has been identified as one of the multiple predictors of poor pregnancy outcome in SLE pregnancies. However, complement activation products, such as Ba, Bb and SC5b-9 might represent more sensitive indicators of complement activation (44).

Complement activation is initiated by classical, alternative, and lectin pathways. The convergence of the three pathways at C3 results in the generation of standard processors: anaphylatoxins, opsonins, and the membrane attack complex. Complement regulatory proteins CD55 (DAF), CD59 (MAC-IP), and CD46 (MCP) are responsible for protecting unregulated complement activation; they interact at different steps of the complement cascade by promoting the inactivation of C3b and C4b and preventing the assembly of C5b-9 (45, 46).

**Figure 2:**
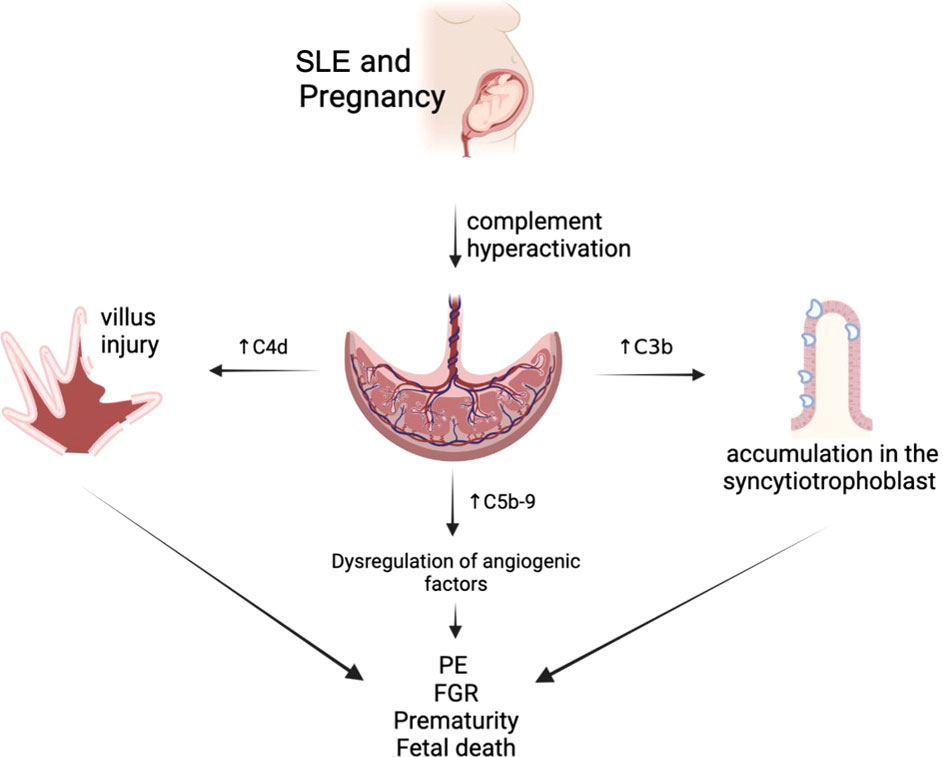
Complement alterations of patients with systemic lupus erythematosus (SLE).

The interpretation of complement levels during pregnancy is uncertain, as circulating complement reflects both synthesis (enhanced by estrogen) and consumption (47). C4 is one of the main components of the classical complement cascade, and C4d, one of its degradation products, binds covalently to cell surface and basement membranes near the site of C4 activation. C4d anchored to the tissue derives in an inflammatory response, which might be related to fetal tissue injury. Therefore, and due to its highly stability and deposition in tissues, acts as a promising marker of classical complement activation and its relation to villous injury (48). Also, the presence of C4d has been associated with FGR cases in patients with PE, SLE, and APS, concurrent with histologic abnormalities in the placenta of these patients (49). In addition, the presence of C4d deposition is associated with a lower gestational age in patients with PE (50) (**Figure 2**).

Increased C5b-9 levels in the first trimester of pregnancy are associated with placental developmental problems and adverse outcomes such as PE due to dysregulation of angiogenic factors (30). This is topic is still under investigation.

Likewise, a retrospective study of 263 patients showed that one-third of patients with adverse pregnancy outcomes had clinical and serologic activity (positive for anti-dsDNA antibodies or low complement) and this finding was associated with fetal loss and prematurity (30).

Recent studies described also the important role of C1q in the physiological vascular remodeling of the decidual spiral artery. C1q is produced and synthesized by extravillous trophoblasts and plays a crucial role in trophoblast invasion of the decidua. Furthermore, in animal experiments, it has been observed that elevated levels of C1q are associated with recurrent losses (51).

Several studies have proposed that women with SLE have impaired placental perfusion either because of excessive activation of the complement system that causes inflammation, or by deficiencies of this system compromising the development and adequate perfusion of uteroplacental unit (52). C3 is the central component of complement system and has been the subject of several genetic studies in pregnancy complications. Functional variants of the C3 gene have been described in patients with idiopathic recurrent miscarriages, it has also been related to the inflammatory process of spiral arteries in patients with PE, and mutations in complement regulators have been described in literature in SLE patients with PE complicated by placental dysfunction (53).

### 3.2 Antiphospholipid antibodies (aPLs)

Another mechanism linking the histological abnormalities and SLE is the embryotoxicity caused by aPLs, which acts on different cellular targets: innate immune cells (neutrophils, monocytes and platelets); endothelial cells and trophoblast cells (1, 29, 54). aPLs are proteins produced by the immune system, directed against different types of phospholipids and phospholipid-binding proteins and are present in up to 30-40% of women with SLE. Lupus anticoagulant (LAC), anticardiolipin antibodies (aCL) and anti-β2 glycoprotein I antibodies (aβ2GPI) are associated with thrombosis, thrombocytopenia and obstetrical morbidity, and are included in the international classification criteria for antiphospholipid syndrome (APS) (55). The relationship of other antibodies, such as anti phosphatidylserine/prothrombin,phosphatidylethanolamine, phosphatidic acid, phosphatidylinositol, vimentin/cardiolipin complex, annexin A5 and IgA antibody isotype anti-β 2 glycoprotein-I and anticardiolipin antibodies with the onset of thrombosis and/or obstetric morbidity is still not well establish, but there is growing evidence suggesting a role for non-criteria aPL in those patients defined as “seronegative” (56). PLs antibodies and its deposition has been observed in the trophoblast and trophoblast basement membranes from SLE patients (29, 54). Placental infarction, impaired spiral artery remodeling, decidua inflammation and the deposition of complement split products were the most common features in placentas of APL-positive women.

Antibodies against β2 glycoprotein I (β2GPI) are localized in trophoblasts and decidual endothelial cells, and they interfere with pregnancy impairing evolution of an adequate placenta development, inhibiting the production of VEGF by endometrial cells and perturbing placental production of growth factor (PlGF). Aβ2GPI autoantibodies also prevent extravillous trophoblast cells function (56–58). B2GPI are deposited in the maternal side of placenta, upholding a harmful imbalance of angiogenic factors and endometrial angiogenesis. They also induce apoptosis in the trophoblast cells, suppress the secretion of chorionic gonadotropin and metalloproteinases, necessary in decidual invasion, and promote the classical pathway of the complement cascade aβ2GPI autoantibodies activate Toll-like receptor (TLR)-4/Myd88 and TLR-8, leading to a proinflammatory state by the production of interleukin (IL)-1β and IL-8 (59, 60).

A decidual vasculopathy/coagulopathy has also been proposed as possible mechanism for SLE and placental antiphospholipid injury. It manifests early in pregnancy and when the damage is severe, it causes extensive placental infarction triggering termination of pregnancy by death or premature delivery; this mechanism is still under study (61).

aPLs bind to the placenta and may induce the decrease of annexin-V (31, 32). Annexin V is a cellular protein known as placental anticoagulant protein. In fact, high levels of annexin V have been found in the placenta (62). Annexin V has a high affinity for anionic phospholipids and therefore interferes with thrombin formation by preventing the binding of activated factor X and prothrombin (63). In a normal pregnancy, annexin V is expressed on apical surfaces of the syncytiotrophoblast microvilli and forms a barrier to phospholipids, and in this way protects against normal coagulation processes (64). The high concentration of annexin V in the trophoblast maintains blood fluidity on the constantly regenerating surfaces of the trophoblast for nutritional exchange between mother, fetus and fetal viability (65). Decrease of annexin V is related to recurrent spontaneous pregnancy loss and placental insufficiency, especially in women with SLE and APS (31, 65). In addition, aPLs suppress annexin V expression in villus trophoblasts and placental villi *in vitro* cultures. This suppression of annexin V has been associated with placental infarction, fetal loss, and recurrent spontaneous pregnancy loss (66). As annexin V and aPL compete for phospholipid binding, this could be an additional mechanism that contributes to placental insufficiency in patients with aPL. The amount of annexin V expressed in the apical membranes of the syncytiotrophoblast is reduced in placentas from patients with APS compared to normal placentas (31, 67, 68).

Regarding aPLs profile associated to adverse pregnancy outcomes, efforts are being made to predict new obstetric complication risks, but such associations are still poorly established today, since most of classifications assess mainly thrombotic manifestations. The PROMISSE study (Predictors of Pregnancy Outcome: bioMarkers In antiphospholipid antibody Syndrome and Systemic lupus Erythematosus) showed that LAC, but not aCL and aβ2GPI was predictive of poor pregnancy outcomes after 12 weeks of pregnancy (69).

Recently, emerging data have raised the question of whether obstetric and vascular APS are the same disease or variants of a syndrome, since a thrombophilic state is a common feature in vascular APS, whereas clot occlusions of the decidual spiral arteries are seldom observed in obstetric APS and infarctions are found in only one-third of APS placentas. In fact, defective placentation is the major cause of pregnancy morbidity in APS, suggesting that non-thrombotic mechanisms might be more important than placental infarction in the pathogenesis of obstetric APS. Tissue distribution of the main target antigen (β2GPI) differs in both variants, being β2GPI expression higher in decidual and placental tissues than in the endothelium might enable higher β2GPI-dependent aPL binding (70).

### 3.3 Neutrophil extracellular traps

Neutrophils are the most predominant type of granulocytes and account for 40% to 70% of all white blood cells in humans. They form an essential part of the innate immune system and are the first line of host defense against invading microorganisms (71). Neutrophil extracellular traps (NETs) is a fibers network composed by chromatin and serine proteases which capture and eliminate extracellular microbes (72). It has been suggested that NETs provide a high local concentration of antimicrobial components and bind, disassemble, and clear microbes independently of phagocytic recruitment. As it was projected, NETs play a role in inflammatory diseases, and they could be detected in PE (73).

NETs are more readily formed in SLE patients, and some are unable to degrade them (74). Jiang et al. have suggested that SLE patients who develop adverse pregnancy outcomes would be associated with inflammatory histological features as well as infiltration of NETs and decidual natural killer cells (dNKs) (2). Other studies have discover that NETs may be involved in aPL-mediated recurrent fetal loss, which is related to inflammatory activation through the complement system (75).

Recently, NETs role has been found as an important fact in the pathogenesis of autoimmune diseases. In SLE, this may result in prolonged exposure to an immune-stimulating context, which exacerbates the autoimmune response and forms a vicious cycle (76, 77). Also, it has have been proposed that NETs play an essential position in the induction and persistence of the vasculopathy described in placentas with SLE and in the occurrence of fetal growth restriction, PE, and fetal loss. It has been described that they are associated with tissue damage and self-antigens production; on the other hand, they have prothrombotic action and proinflammatory action with cytokine production (INF-alpha) (78).

NETs and dNKs are increased in SLE placentas, especially in those with adverse pregnancy outcomes. Neutrophils and NETs presence at the maternal-fetal interface could promote placental damage through the help of dNKs, which develop inflammatory and vascular alterations at entire placenta, thus explaining some of the adverse outcomes observed in SLE patients (79). Increased trophoblast apoptosis has been observed in PE placentas, and the impaired structure and function of spiral arteries have also been described (2). In SLE, an increase in endothelial cell apoptosis has been labelled, which is associated with inflammatory processes characteristic of the disease (80). NETs may be a source of proinflammatory, and antiangiogenic media transmitted to the fetus, with consequences still unknown and understudy.

### 3.4 Cytokines

Most cytokines have proinflammatory characteristics, but some have immunomodulatory or anti-inflammatory functions. Since the pathogenesis of Lupus is not well understood, it is so far unclear whether elevated levels of specific cytokines are driving the disease or just an epiphenomenon of malfunctioning immune regulation and responses, cell death, or elimination of nonviable cellular debris. Nonetheless, some cytokines have been found to correlate with SLE disease activity and have been proposed as therapeutic candidates for active SLE (81, 82).

#### 3.4.1 Interferons type I

IFN are pro-inflammatory cytokines produced in answer to infections as part of host defense by the innate immune system. In the human body, three types of IFNs appear to play a role in SLE: type I, II, and III (83, 84).

Interferon- α has been a critical and the most studied cytokine in the pathogenesis of SLE (85). As is known about half of lupus patients have a predominant expression of interferon-induced genes in their peripheral blood mononuclear cells, which is an enhanced type I interferon gene signature (86). A cohort conducted at Karolinska observed that high IFN-α activity correlates positively with disease activity scores (SLEDAI and SLAM) and with the active involvement of specific organs: e.g., nephritis, arthritis, lymphadenopathy, fatigue, and weight loss. Elevated IFN-α activity is related with SLE disease activity, as it was observed in European and North American cohorts (87, 88).

Nevertheless, its utility in predicting SLE flares and adverse pregnancy has not yet been proven. Regarding the relationship between INF-alpha and adverse perinatal outcomes, elevated interferon-α activity early in gestation has been associated with the onset of PE in SLE patients. In fact, it was found that interferon-α levels are elevated first. Then PE-related symptoms occur in these patients. It is important to remark that women without autoimmune diseases who developed PE did not have increased INF-alpha activity. Andrade et al. described interferon-α induces an antiangiogenic medium, thus increasing the sensitivity of endothelial cells to soluble Flt-1, which suggests that INF-alpha might interfere with the pathogenesis of PE in women with SLE and probably also with other autoimmune diseases (43). Other studies in primary APS suggested a relationship between an enhanced type I IFN gene signature with earlier disease onset and the development of PE (23).

#### 3.4.2 Interferons type II

In the IFN II group, IFN-γ is an antiviral protein generated by mitogen-activated T lymphocytes, and its association with SLE has been described (89). Some studies have characterized IFN-γ levels increase in parallel to the development of autoantibodies and are elevated before disease symptoms were developed. For example, in one cohort of patients, elevated IFN-γ levels were associated with elevated SLEDAI scores, active arthritis, complement consumption, and anti-Ro60/SSA positivity (84, 90).

#### 3.4.3 Interferons type III

The role of type III IFNs, including four subtypes of IFN-λ1, -2, -3, and -4, has recently been identified. IFN-λ is easier to study in SLE patients, as they can be detected in the circulation by conventional ELISA or immunohistochemistry. IFN-λ3 levels have been reported to correlate with SLE disease activity, active NL and arthritis, and supplement consumption (91). Studies have measured IFN-λ levels before and after treatment IFN-λ1 levels decreased only in patients who responded to therapy, while they remained high in histological non-responders. They also observed that elevated IFN-λ1 levels are a feature of patients with cardiovascular events and secondary APS and are often associated with warfarin therapy (92, 93).

Concerning to placentation and pregnancy progress, IFN of all three types play an important role at different stages. The early stages of pregnancy, such as blastocyst implantation and placental invasion, are inflammatory processes that physically degrade and remodel the maternal tissue at the implantation site; in this process, the intervention of type I IFN is indispensable. Subsequent, fetal growth occurs in a Th2 anti-inflammatory environment that typifies most of the pregnancy. Finally, delivery is an inflammatory process in which NF-κβ signaling contributes to labor induction (94).

### 3.5 Chemokines

Chemokines are classified into four subfamilies according to the first two cysteines and the amino acid residues, between them at the N-terminal end of the polypeptide. Chemokines and homeostatic chemokine receptors are essential not just for the arrival of progenitor cells and mature immune cells into primary/secondary immune tissues, also for immune system development and peripheral non-immune tissues, as well as tissue-specific functions and immune surveillance (95). In autoimmune diseases such as SLE, increased expression of chemokine receptors (CXCR2, CXCR3, CCR3 and CCR1) and elevated levels of chemokines such as MCP-1/CCL2, MIP-1/CCL-4, SDF-1/CXCL-12, RANTES/CCL5 and IP-10/CXCL-10 are observed (96). Motta et al. described in their work that CCL5 chemokine was elevated in women with autoimmune diseases; this finding seems to be related to placental villitis and PE (97). However, studies on this topic should be continued, as the evidence and information are limited.

## 4 Conclusion

The placenta is one of the end-organ damaged in SLE in pregnancy. Because its fundamental role in regulating maternal-fetal interactions, its damage is a key pathological pathway leading to adverse pregnancy outcomes in these patients. In recent years, some studies have shredded light into the placental pathophysiologic mechanisms that operate in women with SLE, but, unfortunately, a comprehensive understanding is still elusive. An in-depth knowledge of these mechanisms is required to advance in the prediction, prevention and treatment of the placental damage that mediates association between SLE and adverse perinatal outcomes.

## Author contributions

AC: Manuscript writing, literature review, realization of diagrams and images. NB: Manuscript writing, literature review, manuscript editing. FF: Manuscript editing. DM-P: Manuscript writing, manuscript editing. ES: Manuscript editing. GE: Manuscript editing. All authors contributed to manuscript revision, read, and approved the submitted version.

## Funding

DM-P has been supported by the German Research Foundation (DFG, grant-project nr. Mo2017/3-3- 255955419 and MO2017/4-1 - 468501728 ) and the Interdisciplinary Center for Clinical Research (IZKF, DMMP FF05) at the Jena University Hospital.

## Acknowledgments

The authors declare that the research was conducted in the absence of any commercial or financial relationships that could be construed as a potential conflict of interest.

## Conflict of interest

The authors declare that the research was conducted in the absence of any commercial or financial relationships that could be construed as a potential conflict of interest.

## Publisher’s note

All claims expressed in this article are solely those of the authors and do not necessarily represent those of their affiliated organizations, or those of the publisher, the editors and the reviewers. Any product that may be evaluated in this article, or claim that may be made by its manufacturer, is not guaranteed or endorsed by the publisher.
